# MHD Natural Convection Flow of Casson Nanofluid over Nonlinearly Stretching Sheet Through Porous Medium with Chemical Reaction and Thermal Radiation

**DOI:** 10.1186/s11671-016-1745-6

**Published:** 2016-11-28

**Authors:** Imran Ullah, Ilyas Khan, Sharidan Shafie

**Affiliations:** 1 Department of Mathematical Sciences, Faculty of Science, Universiti Teknologi Malaysia, 81310 UTM Johor Bahru, Johor Malaysia; 2 Basic Sciences Department, College of Engineering, Majmaah University, Majmaah, 11952 Saudi Arabia

**Keywords:** Casson fluid, Chemical reaction, Slip condition, Thermal radiation, Convective boundary condition

## Abstract

In the present work, the effects of chemical reaction on hydromagnetic natural convection flow of Casson nanofluid induced due to nonlinearly stretching sheet immersed in a porous medium under the influence of thermal radiation and convective boundary condition are performed numerically. Moreover, the effects of velocity slip at stretching sheet wall are also examined in this study. The highly nonlinear-coupled governing equations are converted to nonlinear ordinary differential equations via similarity transformations. The transformed governing equations are then solved numerically using the Keller box method and graphical results for velocity, temperature, and nanoparticle concentration as well as wall shear stress, heat, and mass transfer rate are achieved through MATLAB software. Numerical results for the wall shear stress and heat transfer rate are presented in tabular form and compared with previously published work. Comparison reveals that the results are in good agreement. Findings of this work demonstrate that Casson fluids are better to control the temperature and nanoparticle concentration as compared to Newtonian fluid when the sheet is stretched in a nonlinear way. Also, the presence of suspended nanoparticles effectively promotes the heat transfer mechanism in the base fluid.

## Background

Nanofluid is a new class of fluid consists of nanometer-sized particles suspended in a base fluid. Poor heat transfer fluids such as water, ethylene glycol, and engine oil have low thermal conductivity, and are considered essential for heat transfer coefficient between the heat transfer medium and the heat transfer surface. It has been proven through experiments that the thermal conductivity of nanofluid is appreciably higher than the base fluids. The term “nanofluid” was first coined by Choi and Eastman [1] and discovered that suspended nanoparticles in the base fluid can enhance the thermal conductivity of base fluid efficiently. The nanoparticles are typically made of Al_2_O_3_, SiC, AlN, Cu, TiO and graphite, and have high thermal conductivity as compared to conventional base fluids. Eastman et al. [2] further explored that the addition of copper (10 nm) particles in ethylene glycol increases the thermal conductivity up to 40%. Later on, many researchers [3–5] reported that addition of 1–5% by volume of nanoparticles to ordinary heat transfer fluids can enhance the thermal conductivity more than 20%. Boungiorno [6] pointed two slip mechanisms, i.e., Brownian motion and thermophoresis out of seven slip mechanisms that effectively enhance the thermal conductivity of base fluid. Brownian motion is responsible for the collision of nanoparticles moving in the base fluid. In fact, heat transfer due to the collision of two particles could enhance the thermal conductivity of nanofluids. The comprehensive references and in-depth understanding on nanofluid can be insight in most recent articles [7–9].

The boundary layer flow caused by stretching a sheet linearly or nonlinearly is an important engineering problem and has several industrial applications, including extrusion of polymer sheets, melting spinning, the hot rolling, wire drawing, production of glass fiber, plastic and rubber sheets manufacturing, enhanced recovery of petroleum resources, and cooling of large plate in bath. The heat transfer phenomenon in stretching sheet problem is very important as cooling and heating are necessary factors for the quality of end product. The seminal work of Crane [10] was extended by Cortell [11, 12] and found numerical solutions for heat transfer flow of viscous fluid due to nonlinearly stretching sheet with and without the effects of thermal radiation, respectively. In the same year, Abbas and Hayat [13] also explored the influence of thermal radiation on two-dimensional flow of viscous fluid towards nonlinearly stretching sheet saturated in a porous medium. The steady electrically conducting flow of micropolar fluid caused by nonlinearly stretching sheet was reported by Hayat et al. [14]. Motivated by this, Anwar et al. [15] utilized the Boungiorno model and investigated natural convection flow of viscous fluid induced by nonlinearly stretching sheet saturated in a nanofluid. Mukhopadhyay [16] studied two-dimensional boundary layer flow of Casson fluid past a nonlinearly stretching sheet and concluded that fluid velocity is suppressed whereas temperature enhanced by Casson parameter. The two-dimensional incompressible flow of viscous fluid caused by nonlinearly stretching sheet in a nanofluid is reported by Zaimi et al. [17]. Motivate by this, Raju and Sandeep [18] and Raju et al. [19] analyzed three-dimensional electrically conducting flow of Casson-Carreau fluids and nanofluids due to unsteady and steady stretching sheet, respectively. Very recently, Pal et al. [20] investigated the influence of thermal radiation on mixed convection flow of nanofluid caused by nonlinearly stretching/shrinking sheet.

However, the combined effects of heat and mass transfer and chemical reaction play a vital role in chemical and hydro-metallurgical industries. The chemical reaction can be of any order, but the most simple of which is the chemical reaction of first order where the reaction rate and species concentration are directly proportional to each other. The formation of Smog is an example of first order chemical reaction. In several chemical engineering processes, chemical reaction between foreign mass and working fluid often occurs because of stretching a sheet. The diffusive species can be absorbed or generated due to different types of chemical reaction with the ambient fluid which is greatly influenced by the properties and quality of end product. Kandasamy and Periasamy [21] investigated heat and mass transfer free convection flow of Newtonian fluid past nonlinearly stretching sheet in the presence of chemical reaction and magnetic field. The laminar boundary layer flow of electrically conducted fluid towards nonlinearly stretching sheet under the influence of first-order chemical reaction was theoretically studied by Raptis and Perdikis [22]. On the other hand, the numerical and analytical solutions of steady-state boundary layer flow of micropolar fluid induced due to nonlinearly stretching sheet were found by Damseh et al. [23] and Magyari and Chamkha [24], respectively. The combined effects of slip and chemical reaction on electrically conducting fluid over a nonlinearly porous stretching sheet were analyzed by Yazdi et al. [25]. In the same year, Bhattacharyya and Layek [26] investigated the velocity slip effects on boundary layer flow of viscous fluid past a permeable stretching sheet in the presence of chemical reaction. The steady two-dimensional boundary layer flow of Newtonian fluid due to stretching sheet saturated in nanofluid in the presence of chemical reaction is explored by Kameswaran at al. [27]. Motivated by this, Aurangzaib et al. [28] studied theoretically the influence of thermal radiation on unsteady natural convection flow caused by stretching surface in the presence of chemical reaction and magnetic field. Shehzad et al. [29] reported the effects of magnetic field on mass transfer flow of Casson fluid past a permeable stretching sheet in the presence of chemical reaction. Pal and Mandal [30] explored the characteristics of mixed convection flow of nanofluid towards a stretching sheet under the influence of chemical reaction and thermal radiation. Similarity solutions for unsteady boundary flow of Casson fluid induced due to stretching sheet embedded in a porous medium in the presence of first order chemical reaction were obtained by Makanda and Shaw [31].

On the other hand, convective boundary condition plays a vital role in many engineering processes and industries such as gas turbines, material drying, textile drying, laser pulse heating, nuclear plants, transpiration cooling, and food process. It is because that the convective boundary condition applied at the surface is more practical and realistic. The two-dimensional laminar boundary layer flow of Newtonian fluid caused by porous stretching surface in the presence of convective boundary condition is investigated numerically by Ishak [32]. Makinde and Aziz [33] analyzed steady incompressible flow of nanofluids towards stretching sheet with convective boundary condition using Boungiorno model. Moreover, RamReddy et al. [34] included the effects of Soret and investigated mixed convection flow due to vertical plate in a nanofluid under convective boundary condition. Das et al. [35] discussed the heat and mass transfer flow of hydromagnetic nanofluid past a stretching sheet place in a porous medium with convective boundary condition. The three-dimensional laminar flow of Casson nanofluid due to stretching sheet in the presence of convective boundary condition is developed by Nadeem and Haq [36]. Motivated by this, Malik et al. [37] investigated the influence of convective boundary condition past a stretching sheet in the presence of magnetic field. The two-dimensional electrically conducting flow of Casson nanofluid caused by stretching sheet with convective boundary condition is performed by Hussain et al. [38]. Very recently, Sulochana et al. [39] established numerical solutions of three-dimensional Casson nanofluid induced due to permeable stretching sheet in the presence of convective boundary condition.

Motivated by the above-cited literature survey and the widespread engineering and industrial applications, it is of prime importance to explore the effects of chemical reaction and thermal radiation on electrically conducting natural convection flow of Casson nanofluid caused by nonlinearly stretching sheet through porous medium in the presence of slip and convective boundary conditions. The presence of momentum slip and convective boundary condition makes the present mathematical model of a physical system to some extent difficult while interaction of nanofluid with Casson fluid as base fluid. The governing equations are converted to ordinary differential equation using similarity transformations and numerical solutions are obtained through the Keller box method [40]. To validate and examine the numerical algorithm developed in MATLAB software for the present problem, the results are compared with the existing literature results for pure Newtonian and Casson fluids as a limiting case. It is worth mentioning here that the results are perceived in an excellent agreement.

## Methods

### Mathematical Formulation

**Fig. 1:**
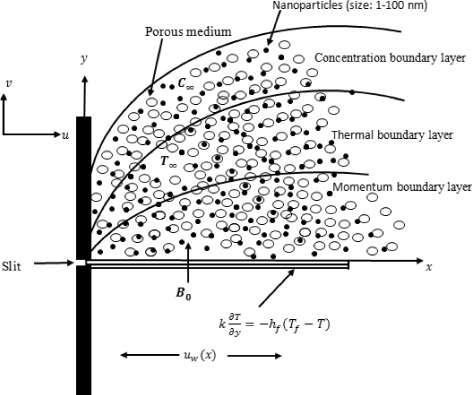
Physical sketch and coordinate system

The steady incompressible natural convection flow of Casson fluid caused by nonlinearly stretching sheet through porous medium in the presence of chemical reaction thermal radiation is considered. The *x* − axis is taken along the direction of stretching sheet and *y* − axis is perpendicular to the surface (see Fig. 1). The sheet is stretched with the nonlinear velocity of the form *u*_*w*_(*x*) = *cx* ^*n*^, where *c* is constant and *n* (> 0) represents the nonlinearly stretching sheet parameter (*n* = 1 corresponds to linear stretching sheet and *n* ≠ 1 represent nonlinear stretching sheet). Moreover, a variable magnetic field *B*(*x*) = *B*_0_ *x* ^(*n* − 1)/2^ [22] is applied normally to the stretching sheet with constant *B*_0_. Furthermore, it is also assumed that sheet wall is heated by temperature *T*_*f*_(*x*) = *T*_∞_ + *Ax* ^*λ*^ (*A* being the reference temperature and *λ* = 2*n* − 1) and *C*_*s*_(*x*) = *C*_∞_ + *Ex* ^*λ*^ (*E* being the reference concentration).

The governing equations for Casson nanofluid along with continuity equation are given as

1 $$ \frac{\partial u}{\partial x}+\frac{\partial \upsilon }{\partial x}=0, $$

$$ u\frac{\partial u}{\partial x}+\upsilon \frac{\partial u}{\partial y}=\frac{\mu_B}{\rho_f}\left(1+\frac{1}{\beta}\right)\;\frac{\partial^2u}{\partial {y}^2}-\left(\frac{\sigma {B}^2(x)}{\rho_f}+\frac{\mu_B\phi }{\rho_f{k}_1}\right)u $$

2 $$ +\left[\left(1-{C}_{\infty}\right)\frac{\rho_{f_{\infty }}}{\rho_f}{\beta}_T\left(T-{T}_{\infty}\right)+\frac{\left({\rho}_p-{\rho}_{f_{\infty }}\right)}{\rho_f}g{\beta}_C\left(C-{C}_{\infty}\right)\right]g, $$

3 $$ u\frac{\partial T}{\partial x}+\upsilon \frac{\partial T}{\partial y}={\alpha}_f\frac{\partial^2T}{\partial {y}^2}+\tau \left[{D}_B\frac{\partial C}{\partial y}\frac{\partial T}{\partial y}+\frac{D_T}{T_{\infty }}{\left(\frac{\partial T}{\partial y}\right)}^2\right]-\frac{1}{{\left(\rho c\right)}_f}\frac{\partial {q}_r}{\partial y}, $$

4 $$ u\frac{\partial C}{\partial x}+\upsilon \frac{\partial C}{\partial y}={D}_B\frac{\partial^2C}{\partial {y}^2}+\frac{D_T}{T_{\infty }}\frac{\partial^2T}{\partial {y}^2}-{k}_c\left(C-{C}_{\infty}\right) $$

In the above expressions, *u* and *υ* denote the velocity components in *x* − and *y* − directions, respectively, *μ*_*B*_ is the plastic dynamic viscosity, *ρ*_*f*_ is the fluid density, *σ* is the electrically conductivity, *β* is the Casson fluid parameter which has inverse relation with yield stress, i.e., ${p}_y=\frac{\mu_B\sqrt{2\pi }}{\beta }$ [41], *φ* is the porosity, *k*_1_(*x*) = *k*_0_ *x* ^1 − *n*^ is the variable permeability of porous medium, *g* is the gravitational force due to acceleration, *ρ*_*p*_ is the density of nanoparticle, *β*_*T*_ is the volumetric coefficient of thermal expansion, *β*_*C*_ is the coefficient of concentration expansion, *T* is the fluid temperature, *C* is the nanoparticle concentration, ${\alpha}_f=\frac{k}{{\left(\rho c\right)}_f}$ is the thermal diffusivity of the Casson fluid, *k* is the thermal conductivity of the fluid, *D*_*B*_ is the Brownian diffusion coefficient, *D*_*T*_ is the thermophoretic diffusion coefficient, $\tau =\frac{{\left(\rho c\right)}_p}{{\left(\rho c\right)}_f}$ is the ratio of heat capacities in which (*ρc*)_*f*_ is the heat capacity of the fluid and (*ρc*)_*p*_ is the effective heat capacity of nanoparticle material, *c*_*p*_ is the specific heat at constant pressure, *q*_*r*_ is the radiative heat flux and ${k}_c(x)=\frac{a{k}_2{x}^n}{x}$ is the variable rate of chemical reaction, *k*_2_ is a constant reaction rate, and *a* is the reference length along the flow.

The corresponding boundary conditions are written as follows:

5 $$ \left.\begin{array}{l}u={u}_w(x)+{N}_1\nu \left(1+\frac{1}{\beta}\right)\frac{\partial u}{\partial y}, k\frac{\partial T}{\partial y}=-{h}_f\left({T}_f-T\right)\ \\ {}{D}_B\frac{\partial C}{\partial y}=-{h}_s\left({C}_s-C\right)\kern1em \mathrm{at}\kern0.5em y=0\ \end{array}\right\}, $$

6 $$ u\to 0,\kern0.5em T\to {T}_{\infty },\ C\to {C}_{\infty }\ \mathrm{a}\mathrm{s}\kern1em y\to \infty . $$

Here, ${N}_1(x)={N}_0{x}^{-\frac{n-1}{2}}$ is the velocity slip, *N*_0_ being constant, ${h}_f(x)={h}_0{x}^{\frac{n-1}{2}}$ and ${h}_s(x)={h}_1{x}^{\frac{n-1}{2}}$ are the convective heat and mass transfer with *h*_0_, and *h*_1_ being constants.

The radiative heat flux *q*_*r*_ described according to Rosseland approximation is given as

7 $$ {q}_r=\frac{-4{\sigma}^{\ast }}{3{k_1}^{*}}\frac{\partial {T}^4}{\partial y} $$

where *σ* ^∗^ is the Stefan-Boltzmann constant and *k*_1_* is the mean absorption coefficient. *T* ^4^ can be expressed as linear function of temperature. By expanding *T* ^4^ in a Taylor series about *T*_∞_ and neglecting higher terms, we can write

8 $$ {T}^4\cong 4{T}_{\infty}^3T-3{T}_{\infty}^4 $$

Now putting Eqs. (7) and (8) in Eq. (3), we obtain

9 $$ u\frac{\partial T}{\partial x}+\upsilon \frac{\partial T}{\partial y}={\alpha}_f\frac{\partial^2T}{\partial {y}^2}+\frac{16{\sigma}^{\ast }{T}_{\infty}^3}{3{\left(\rho c\right)}_f{k_1}^{*}}\frac{\partial^2T}{\partial {y}^2}+\tau \left[{D}_B\frac{\partial C}{\partial y}\frac{\partial T}{\partial y}+\frac{D_T}{T_{\infty }}{\left(\frac{\partial T}{\partial y}\right)}^2\right] $$

Now, introduce the stream function *ψ* defined in its usual notation in terms of velocity, a similar variable *η*, and the following similarity transformations;

10 $$ \psi =\sqrt{\frac{2\nu c}{n+1}}{x}^{\frac{n+1}{2}}f\left(\eta \right),\kern0.37em \eta =\sqrt{\frac{\left(n+1\right)c}{2\nu }}{x}^{\frac{n-1}{2}}y,\kern0.37em \theta =\frac{T-{T}_{\infty }}{T_f-{T}_{\infty }}, \varphi =\frac{C-{C}_{\infty }}{C_s-{C}_{\infty }} $$

Finally, Eqs. (3–6) and Eq. (9) take the following form

11 $$ \left(1+\frac{1}{\beta}\right)\;{f}^{{\prime\prime} }+f{f}^{{\prime\prime} }-\frac{2n}{n+1}{f^{\prime}}^2-\frac{1}{n+1}\left(M+K\right){f}^{\prime }+\frac{1}{n+1}\left(\mathrm{G}\mathrm{r}\theta +\mathrm{Gm}\varphi \right)=0 $$

12 $$ \left(1+\frac{4}{3}{R}_d\right)\;{\theta}^{{\prime\prime} }+ \Pr f{\theta}^{\prime }-\frac{2\left(2n-1\right)}{n+1} \Pr {f}^{\prime}\theta + \Pr {N}_b{\varphi}^{\prime }{\theta}^{\prime }+ \Pr {N}_t{\theta^{\prime}}^2=0 $$

13 $$ {\varphi}^{{\prime\prime} }+Lef{\varphi}^{\prime }-\frac{2\left(2n-1\right)}{n+1}Le{f}^{\prime}\varphi +\frac{N_t}{N_b}{\theta}^{{\prime\prime} }-\frac{1}{n+1} RLe\varphi =0 $$

14 $$ \left.\begin{array}{l}{f}^{\prime }(0)=1+\delta \sqrt{n+1}\left(1+\frac{1}{\beta}\right){f}^{{\prime\prime} }(0),\kern0.62em {\theta}^{\prime }(0)=-\left(\operatorname{},\sqrt{\frac{1}{n+1}}\right)B{i}_1\left[1-\theta (0)\right],\kern0.5em \\ {}{\varphi}^{\prime }(0)=-\left(\sqrt{\frac{1}{n+1}}\right)B{i}_2\left[1-\varphi (0)\right]\end{array}\right\} $$

15 $$ {f}^{\prime}\left(\infty \right)=0,\kern1.62em \theta \left(\infty \right)=0,\kern1em \phi \left(\infty \right)=0 $$

In the above expressions, *M*, *K*, Gr, Gm, *δ*, Pr, *R*_*d*_, *N*_*b*_, *N*_*t*_, *Bi*_1_, *Bi*_2_, Le, and *R* are the magnetic parameter, porosity parameter, Grashof number, mass Grashof number, slip parameter, Prandtl number, radiation parameter, Brownian motion parameter, thermophoresis parameter, Biot numbers, Lewis number, and chemical reaction parameter and are defined as

$$ \begin{array}{l}M=\frac{2\sigma {B}_0^2}{\rho c}\kern0.62em ,K=\frac{2\nu \phi }{k_0c}\kern0.62em ,\mathrm{G}\mathrm{r}=\frac{2g{\beta}_TA\left(1-{C}_{\infty}\right)\left(\raisebox{1ex}{${\rho}_{f_{\infty }}$}\!\left/ \!\raisebox{-1ex}{$\rho f$}\right.\right)}{c^2},\mathrm{G}\mathrm{m}=\frac{2g{\beta}_CE\left(\raisebox{1ex}{$\left({\rho}_p-{\rho}_{f_{\infty }}\right)$}\!\left/ \!\raisebox{-1ex}{${\rho}_f$}\right.\right)}{c^2},\\ {}\delta ={N}_0\sqrt{\frac{c}{2\nu }}, \Pr =\frac{\mu {c}_f}{k},{R}_d=\frac{4{\sigma}^{\ast }{T}_{\infty}^3}{k{k}_1^{*}},{N}_b=\frac{\tau {D}_B\left({C}_s-{C}_{\infty}\right)}{\nu },{N}_t=\frac{\tau {D}_T\left({T}_f-{T}_{\infty}\right)}{\nu {T}_{\infty }},\\ {}{\mathrm{Bi}}_1=\frac{h_0}{k}{\left[\frac{2\nu }{c}\right]}^{\frac{1}{2}},{\mathrm{Bi}}_2=\frac{h_1}{D_B}{\left[\frac{2\nu }{c}\right]}^{\frac{1}{2}},\mathrm{L}\mathrm{e}=\frac{\nu }{D_B},R=\frac{2\nu {k}_2}{c}\end{array} $$

The wall skin friction, wall heat flux, and wall mass flux, respectively, are defined by

$$ {\tau}_w={\mu}_B\left(1+\frac{1}{\beta}\right)\kern0.28em {\left[\frac{\partial u}{\partial y}\right]}_{y=0},\kern0.62em {q}_w=-{\left(\left({\alpha}_f+\frac{16{\sigma}^{\ast }{T}_{\infty}^3}{3{\left(\rho c\right)}_f{k_1}^{\ast }}\right)\kern0.28em \frac{\partial T}{\partial y}\right)}_{y=0}\mathrm{and}\kern0.5em {q}_s=-{D}_B{\left(\frac{\partial C}{\partial y}\right)}_{y=0} $$

The dimensionless skin friction coefficient $C{f}_x=\frac{\tau_w}{\rho {u}_w^2}$, the local Nusselt number $N{u}_x=\frac{x{q}_w}{\alpha_f\left({T}_f-{T}_{\infty}\right)}$, and local Sherwood number $S{h}_x=\frac{x{q}_s}{D_B\left({C}_w-{C}_{\infty}\right)}$ on the surface along *x* − direction, local Nusselt number *Nu*_*x*_, and Sherwood number *Sh*_*x*_ are given by

$$ \begin{array}{l}{\left(R{e}_x\right)}^{1/2}C{f}_x=\sqrt{\frac{n+1}{2}}\left(1+\frac{1}{\beta}\right){f}^{{\prime\prime} }(0),\kern1.12em {\left(R{e}_x\right)}^{-1/2}N{u}_x=-\sqrt{\frac{n+1}{2}}\left(1+\frac{4}{3}{R}_d\right){\theta}^{\prime }(0),\\ {}{(Re)}^{-1/2}S{h}_x=-\sqrt{\frac{n+1}{2}}{\varphi}^{\prime }(0)\;\end{array} $$

where $R{e}_x=\frac{c{x}^{n+1}}{\nu }$ is the local Reynold number.

### Numerical Scheme

The governing Eqs. (11)–(13) with associated boundary conditions (14) and (15) are solved numerically via the Keller box method. The detail of this method is given in the book of Cebeci and Bradshaw [40]. This method is unconditionally stable and has second-order accuracy. The following four steps are involved in finding the numerical solutions of the problem

(i) Initially, the transformed governing equations are converted to first-order system.
(ii) Now, approximate the first-order system using central difference formula about the mid-point.
(iii) The algebraic equations are then linearized via Newton’s method and write them in matrix-vector notation.
(iv) Finally, block tri-diagonal elimination technique is used to solve the linear system.

Here, the step size *η* = 0.01 and boundary layer thickness *η*_∞_ = 10 is used. Further, the convergent criteria 10^− 5^ is considered for all the cases. The numerical and graphical results are generated through MATLAB software. In order to assess the accuracy and validate our code, the comparison is made with previous results of literature as a limiting case.

## Results and Discussion

In the present study, the effects of slip and convective boundary conditions on heat and mass transfer flow of nanofluid due to nonlinearly stretching surface saturated in a porous medium in the presence of chemical reaction and thermal radiation were analyzed. Moreover, Casson fluid is used as base fluid. In order to analyze the results, numerical calculations are carried out for various values of Casson fluid parameter *β*, nonlinear stretching sheet parameter *n*, magnetic parameter *M*, porosity parameter *K*, Grashof number *Gr*, mass Grashof number *Gm*, Prandtl number Pr, radiation parameter *R*_*d*_, Brownian motion parameter *N*_*b*_, thermophoresis parameter *N*_*t*_, Lewis number *Le*, slip parameter *δ*, and Biot numbers *Bi*_1_, *Bi*_2_. For validation of the present method, the results are compared with previously reported results and displayed in Tables 1, 2, and 3.

**Table 1:** Comparison of skin friction coefficient for different values of *β* and *M* when *n* = 1, *β* → ∞, *Bi*_1_ → ∞, *Bi*_2_ → ∞, and *M* = *K* = Gr = Gm = *δ* = *R*_*d*_ = Le = *N*_*t*_ = *N*_*b*_ = *R* = 0

$$ -\left(1+\frac{1}{\beta}\right){f}^{{\prime\prime} }(0) $$				
*β*	*M*	Nadeem et al. [36]	Ahmad and Nazar [42]	Present results
∞	0	1.0042	1.0042	1
5		1.0954	–	1.0955
1		1.4142	–	1.4144
∞	10	3.3165	3.3165	3.3166
5		3.6331	–	3.6332
1		4.6904	–	4.6904
∞	100	10.049	10.0498	10.0499
5		11.0091	–	11.0091
1		14.2127	–	14.2127

**Table 2:** Comparison of skin friction coefficient when *β* → ∞, *Bi*_1_ → ∞, *Bi*_2_ → ∞, Pr = 6.8, and *M* = *K* = Gr = Gm = *δ* = *R*_*d*_ = Le = *N*_*t*_ = *N*_*b*_ = *R* = 0

$$ -\left(1+\frac{1}{\beta}\right){f}^{{\prime\prime} }(0) $$			
*n*	Cortell [11]	Pal et al. [20]	Present results
0.0	0.6276	0.6275	0.6276
0.2	0.7668	0.7668	0.7668
0.5	0.8895	0.8895	0.8896
1	1.0000	1.0000	1.0000
3	1.1486	1.1486	1.1486
10	1.2349	1.2348	1.2349
20	1.2574	1.2574	1.2574

**Table 3:** Comparison of − *θ*′(0) for different Pr with *n* = 1, *β* → ∞, *Bi*_1_ → ∞, *Bi*_21_ → ∞, and *M* = *K* = Gr = Gm = *δ* = *R*_*d*_ = Le = *N*_*t*_ = *N*_*b*_ = *R* = 0

− *θ*′(0)				
Pr	Yih [43]	Aurangzaib et al. [28]	Pal et al. [20]	Present results
0.72	0.8086	0.8086	0.8086	0.8088
1	1.0000	1.0000	1.0000	1.0000
3	1.9237	1.9237	1.9237	1.9237
10	3.7207	3.7207	3.7206	3.7208
100	12.2940	12.3004	12.2939	12.3004

Tables 1 and 2 present the comparison of skin friction coefficient for different values of *β*, *M*, and *n*, respectively, with the results of Nadeem et al. [36], Ahmad and Nazar [42], Cortell [11], and Pal et al. [20]. The results showed an excellent agreement. Table 3 describes the comparison of heat transfer rate for various values of Pr with the results of Yih [43], Aurangzaib et al. [28], and Pal et al. [20] and revealed in a good agreement.

**Fig. 2:**
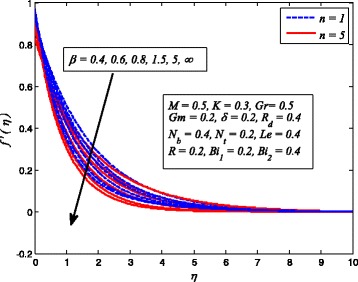
Effect of *β* on velocity for two different values of *n*

**Fig. 3:**
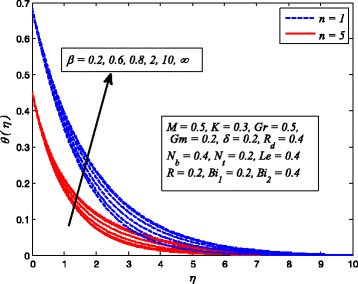
Effect of *β* on temperature for various *n*

**Fig. 4:**
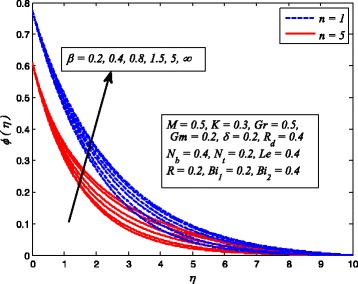
Effect of *β* on nanoparticle concentration for two different values of *n*

**Fig. 5:**
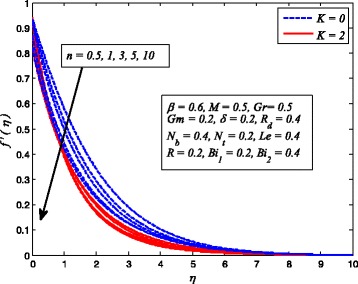
Effect of *n* on velocity in the presence/absence of *K*

**Fig. 6:**
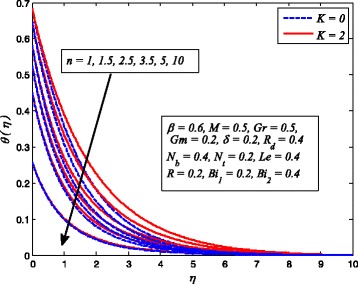
Effect of *n* on temperature in the presence/absence of *K*

**Fig. 7:**
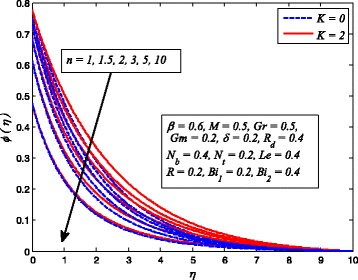
Effect of *n* on nanoparticle concentration in the presence/absence of *K*

**Fig. 8:**
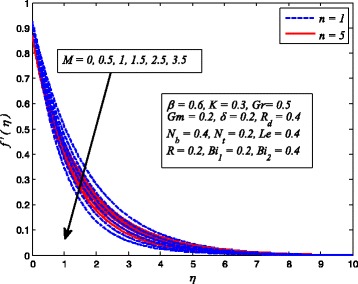
Effect of *M* on velocity for various values of *n*

**Fig. 9:**
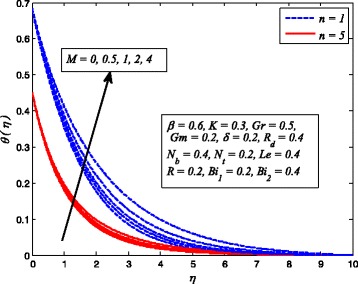
Effect of *M* on temperature for two values of *n*

**Fig. 10:**
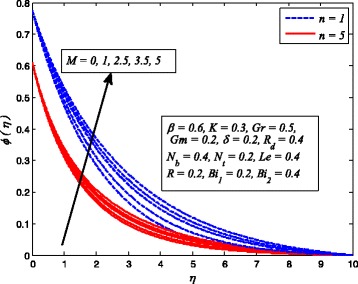
Effect of *M* on nanoparticle concentration for two values of *n*

**Fig. 11:**
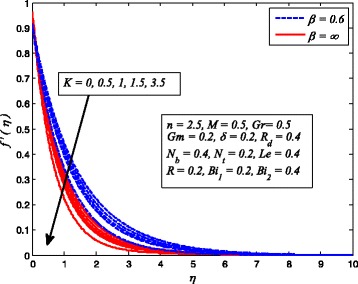
Effect of *K* on velocity for various values of *β*

**Fig. 12:**
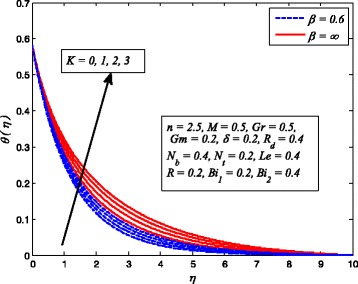
Effect of *K* on temperature for two values of *β*

**Fig. 13:**
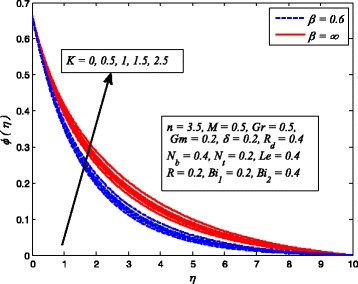
Effect of *K* on nanoparticle concentration for various *β*

**Fig. 14:**
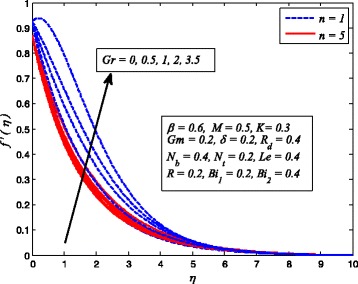
Effect of *Gr* on velocity for two different values of *n*

**Fig. 15:**
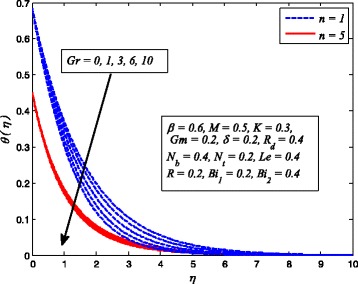
Effect of *Gr* on temperature profile for two different values of *n*

**Fig. 16:**
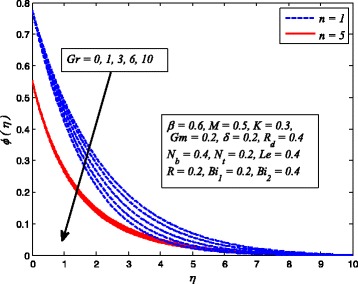
Effect of *Gr* on nanoparticle concentration for various *n*

**Fig. 17:**
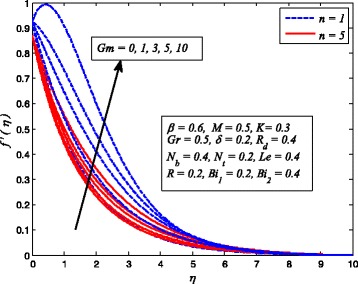
Effect of *Gm* on velocity for two different values of *n*

**Fig. 18:**
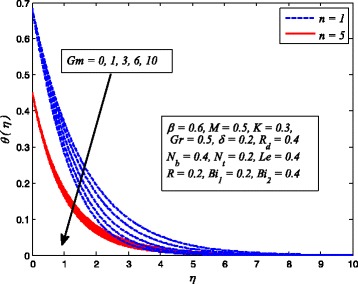
Effect of *Gm* on temperature for two values of *n*

**Fig. 19:**
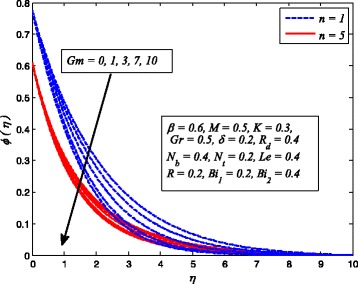
Effect of *Gm* on nanoparticle concentration for different values of *n*

**Fig. 20:**
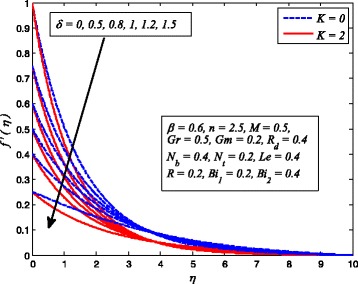
Effect of *δ* on velocity in the presence/absence of *K*

**Fig. 21:**
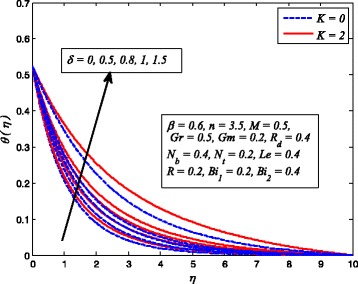
Effect of *δ* on temperature in the presence/absence of *K*

**Fig. 22:**
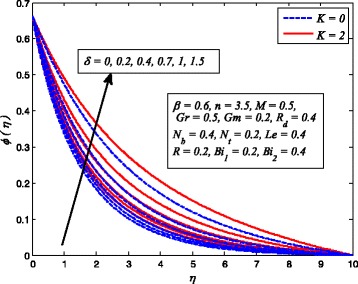
Effect of *δ* on nanoparticle concentration in the presence/absence of *K*

**Fig. 23:**
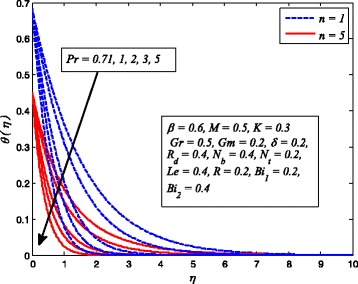
Effect of Pr on temperature profile for various *n*

**Fig. 24:**
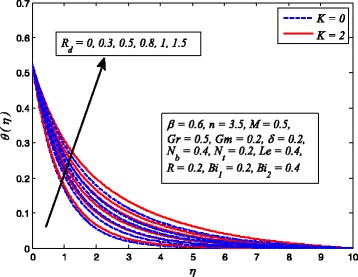
Effect of *R*_*d*_ on temperature profile in the presence/absence of *K*

**Fig. 25:**
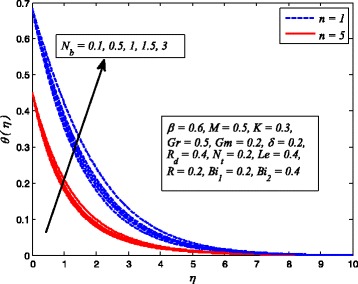
Effect of *N*_*b*_ on temperature profile for different *n*

**Fig. 26:**
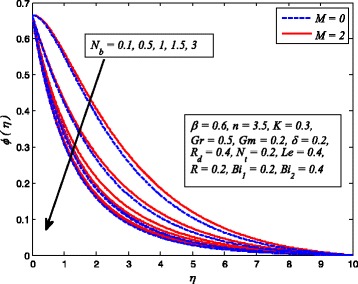
Effect of *N*_*b*_ on nanoparticle concentration for two different values of *M*

**Fig. 27:**
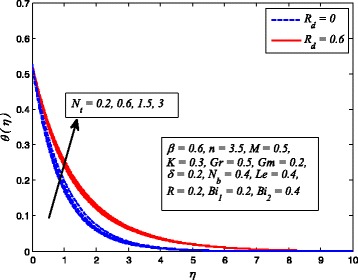
Effect of *N*_*t*_ on temperature profile in the presence/absence of *R*_*d*_

**Fig. 28:**
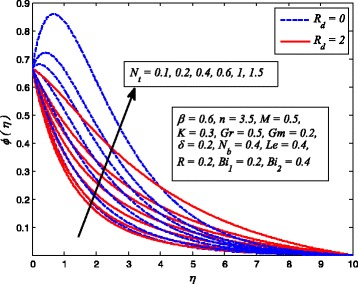
Effect of *N*_*t*_ on nanoparticle concentration in the presence/absence of *R*_*d*_

**Fig. 29:**
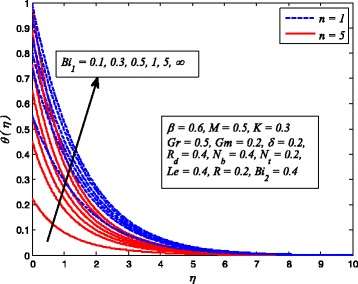
Effect of *Bi*_1_ on temperature profile for various *n*

**Fig. 30:**
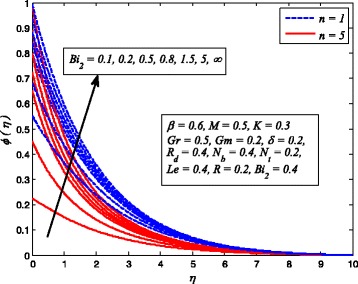
Effect of *Bi*_2_ on nanoparticle concentration for various *n*

**Fig. 31:**
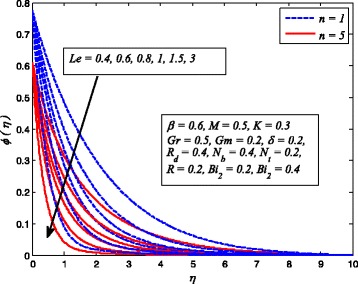
Effect of *Le* on nanoparticle concentration for various values of *n*

Figures 2, 3, 4, 5, 6, 7, 8, 9, 10, 11, 12, 13, 14, 15, 16, 17, 18, 19, 20, 21, 22, 23, 24, 25, 26, 27, 28, 29, 30, and 31 are displayed to insight the physical behavior of *β*, *n*, *M*, *K*, *Gr*, *Gm*, *δ*, Pr, *R*_*d*_, *N*_*b*_, *N*_*t*_, *Bi*_1_, *Bi*_2_, *Le*, and *R* on velocity (*f*′(*η*)), temperature (*θ*(*η*)), and nanoparticle concentration (*φ*(*η*)) profiles, respectively. Figures 2, 3, and 4 exhibit the variation of *β* on velocity, temperature, and nanoparticle concentration, respectively, for the case of *n* = 1 and *n* ≠ 1. It is noteworthy here that the present problem reduces to pure Newtonian nanofluid case when *β* → ∞. Clearly, fluid velocity reduces as *β* increases. The reason behind this behavior is that increasing values of *β* implies rise in fluid viscosity, i.e., reducing the yield stress. Consequently, the momentum boundary layer thickness reduces. It is also observed from this figure that fluid velocity decreases faster in the case of stretching a sheet in a nonlinear way. A similar trend of velocity profile was reported by Nadeem and Haq [36] and Mukhopadhyay [16]. Conversely, both *θ*(*η*) and *φ*(*η*) enhance with increase in *β* (see Figs. 3 and 4). Figures 5, 6, and 7 demonstrate the effect of *n* on fluid velocity, temperature, and nanoparticle concentration, respectively, in non-porous (*K* = 0) and porous (*K* ≠ 0) medium. Interestingly, *f*′(*η*), *θ*(*η*), and *φ*(*η*) are all found as decreasing functions of *n*. It is also noticed from Fig. 5 that momentum boundary layer thickness reduces rapidly when *K* ≠ 0, whereas the case of thermal and concentration boundary layer thicknesses are quite opposite to this, i.e., both thicknesses decrease when *K* = 0.

Figures 8, 9, and 10 reveal the influence of *M* on fluid velocity, temperature, and nanoparticle concentration, respectively, for both cases of *n* = 1 and *n* ≠ 1. It is noticeable that increasing values of *M* reduces the fluid velocity whereas temperature and nanoparticle concentration rises as *M* increases. As it is well-known fact that a resistive-type force produces as the current passes through the moving fluid. This force is responsible in slowing down fluid motion and increasing the thermal and concentration boundary layer thicknesses. A similar behavior for electrically conducting flow of Casson nanofluid due to stretching sheet was observed by Hussain et al. [38].

The effect of *K* on velocity, temperature, and nanoparticle concentration profiles for Newtonian and non-Newtonian fluids is portrayed in Figs. 11, 12, and 13, respectively. It is interesting to note that the response of *K* in these figures is completely the same as observed for *M*. It is also found from these figures that momentum boundary layer become thinner in case of *β* → ∞ and the opposite to this for thermal and concentration boundary layer thicknesses.

Figures 14, 15, and 16 display the variation of *Gr* velocity, temperature, and nanoparticle concentration distributions for *n* = 1 and *n* ≠ 1. It is seen that fluid velocity rises with increase in *λ*_*T*_ whereas temperature and nanoparticle concentration reduce as *Gr* increases. Since the buoyancy force is dominant over viscous force with increase in *Gr*. Consequently, Grashof number enhances the fluid flow which leads to increasing velocity as well as thickness of momentum boundary layer. In addition, since the buoyancy force tends to enhance the temperature and concentration gradients, therefore, temperature and nanoparticle concentration fall. The same reason may be described for the behavior of *Gm* on velocity, temperature, and nanoparticle concentration distributions, as elucidated in Figs. 17, 18, and 19. It is also observed from these figures that influence of *Gr* and *Gm* is more pronounced in case of linear stretching sheet for all the three profiles.

The variations of *δ* on dimensionless velocity, temperature, and nanoparticle concentration profiles for *K* = 0 and *K* ≠ 0 are depicted in Figs. 20, 21, and 22, respectively. It is worth mentioning here that *δ* = 0 corresponds to no slip condition and *δ* ≠ 0 shows velocity slip at stretching sheet wall. It is interesting to see that increasing values of *δ* reduces the fluid velocity initially and then increases far from the sheet whereas dimensionless temperature and nanoparticle concentration increase with increase in *δ*. Physically, this shows that fluid velocity adjacent to the sheet is less than the velocity of normal stretching sheet as slip (*δ* ≠ 0) occurs. Increasing *δ* allowed more fluid slipping over the sheet and the flow decelerates near the sheet.

Figure 23 reveals the influence of Pr on dimensionless temperature profile when *n* = 1 and *n* ≠ 1. As expected, increasing Pr leads to reduction in dimensionless temperature. Based on the definition of Pr (the ratio of momentum diffusivity to thermal diffusivity), therefore, for large Pr, heat will diffuse more rapidly than the momentum. Consequently, thickness of thermal boundary layer reduces as Pr increases. It is also noticed that higher values of Pr reduces the temperature more drastically. It is because of the fact that low thermal conductivity of fluid associated with larger Pr, which decreases conduction and results a temperature fall.

The effect of *R*_*d*_ on dimensionless temperature for *K* = 0 and *K* ≠ 0 is exhibited in Fig. 24. It is noteworthy here that *R*_*d*_ = 0 denotes no radiation and *R*_*d*_ ≠ 0 shows the presence of radiation. Clearly, dimensionless temperature is higher as *R*_*d*_ increases. The reason behind this fact is that heat energy released to the fluid as *R*_*d*_ increases and this results rise in temperature. It is also evident from this figure that thermal boundary layer thickness increases faster with increase in *R*_*d*_ when *K* ≠ 0. This shows that influence of radiation in a porous medium is more effective when high temperature is required for the desired thickness of end product.

Figures 25 and 26 illustrate the variation of *N*_*b*_ on dimensionless temperature and nanoparticle concentration distributions. It is noted that dimensionless temperature enhances with increase in *N*_*b*_ while nanoparticle concentration is found decreasing as *N*_*b*_ increases. It is well known that Brownian motion is a diffusive process. The higher diffusivity implies higher temperature, and as consequences, the thermal conductivity becomes higher. Also, Brownian motion in nanofluid occurs only due to nanometer size of nanoparticles. In addition to this, the kinetic energy of nanoparticles enhance mainly due to the increase in *N*_*b*_ and resulting higher temperature of nanofluids. The influence of *N*_*t*_ on dimensionless temperature and nanoparticle concentration distributions for *R*_*d*_ = 0 and *R*_*d*_ ≠ 0 are shown in Figs. 27 and 28, respectively. It is evident from these figures that both *θ*(*η*) and *φ*(*η*) are increasing functions of *N*_*t*_. According to the definition of *N*_*t*_, i.e., higher values of *N*_*t*_ implies higher temperature differences and shear gradient. Therefore, increasing values of *N*_*t*_ tends to higher temperature difference across the boundary layer. On the other hand, nanoparticle concentration is a strong function of *N*_*t*_; for this reason, it is significantly influenced by increasing values of *N*_*t*_. These conclusions are in agreement with Hussain et al. [38] and Nadeem et al. [36]. It is also interesting to note from Fig. 28 that concentration peak values reveal that stronger *N*_*t*_ intensifies the thermal conductivity of the nanofluids near the wall. Further, it is also observed from Fig. 28 that nanoparticle concentration falls with increase in *R*_*d*_.

Figures 29 and 30 demonstrate the effect of *Bi*_1_ and *Bi*_2_ on dimensionless temperature and nanoparticle concentration profiles for *n* = 1 and *n* ≠ 1. It is worth mentioning that the present study will reduce to constant wall temperature and constant wall concentration case when *Bi*_1_ → ∞ and *Bi*_2_ → ∞. Apparently, dimensionless temperature increases with increase in *Bi*_1_. It is also evident from this figure that increasing values of *Bi*_1_ lead to rise in sheet convective heating. Furthermore, smaller values of *Bi*_1_ (< < 1) rapidly enhance the temperature as well as the corresponding boundary layer thickness across the boundary region. Since *Bi*_2_ is inversely proportional to Brownian diffusivity coefficient. Therefore, thermal diffusivity reduces whereas momentum diffusivity rises, and as a consequence, nanoparticle concentration enhances and related boundary layer becomes thinner.

**Fig. 32:**
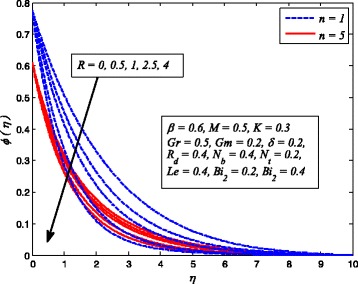
Effect of *R* on nanoparticle concentration for two different values of *n*

Figure 31 exhibits the variation of *Le* on nanoparticle concentration profile for linear and nonlinear stretching sheet. It is perceived that in both cases, nanoparticle concentration is lower for higher *Le*. It is a fact that lower *D*_*B*_ corresponds to larger *Le* and the fluids having smaller *Le* have higher *D*_*B*_. In other words, mass transfer rate higher for large *Le*. The influence of *R* on nanoparticle concentration distribution for *n* = 1 and *n* ≠ 1 is depicted in Fig. 32. It is noteworthy here that *R* = 0 denotes no chemical reaction and *R* ≠ 0 corresponds to the presence of chemical reaction. It is evident that stronger *R* leads to reduce nanoparticle concentration. The explanation for this behavior is that destructive chemical rate (*R* > 0) enhances the mass transfer rate and results a decrease in nanoparticle concentration.

**Fig. 33:**
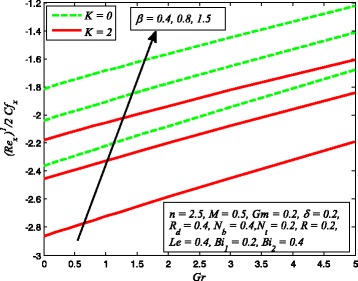
Variation of skin friction coefficient for various values of *β*, *Gr*, and *K*

**Fig. 34:**
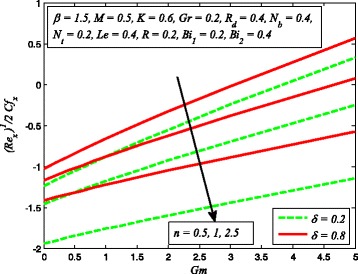
Variation of skin friction coefficient for different values of *δ*, *n*, and *Gm*

**Fig. 35:**
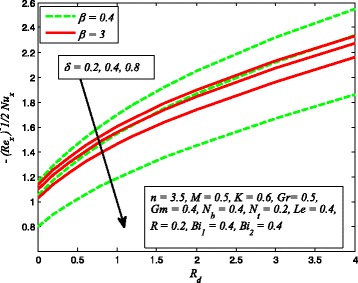
variation of Nusselt number for various values of *R*_*d*_, *β*, and *δ*

**Fig. 36:**
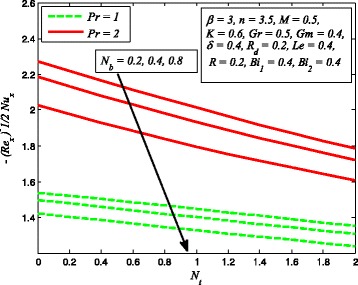
Variation of Nusselt number for various values of *N*_*t*_, *N*_*b*_, and Pr

**Fig. 37:**
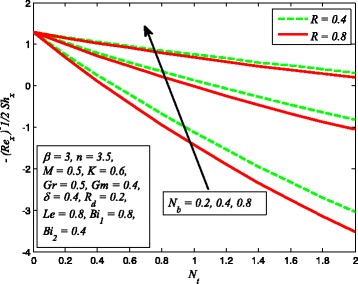
Variation of Sherwood number for various values of *N*_*t*_, *N*_*b*_, and *R*

**Fig. 38:**
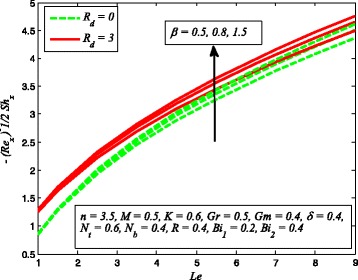
variation of Sherwood number for various values of *β*, *Le*, and *R*_*d*_

The effect of skin friction coefficient *Cf*_*x*_, local Nusselt number *Nu*_*x*_, and Sherwood number *Sh*_*x*_ for some physical parameters *β*, *n*, *K*, *Gr*, *Gm*, *δ*, Pr, *R*_*d*_, *N*_*b*_, *N*_*t*_, *Le*, and *R* are displayed in Figs. 33, 34, 35, 36, 37, and 38, respectively. Figure 33 reveals the variation of wall shear stress for increasing values of *β*, *Gr*, and *K*. Clearly, $$ \left|\sqrt{\frac{n+1}{2}}\left(1+\raisebox{1ex}{$1$}\!\left/ \!\raisebox{-1ex}{$\beta $}\right.\right){f}^{{\prime\prime} }(0)\right| $$ decreases with increase in *β* and *Gr*, whereas it increases with increase in *K*. It is also evident from this figure that the skin friction coefficient is negative for all values of *β*, *Gr*, and *K* which indicates that fluid experiences a resistive force at the boundary. This is also in agreement with the results of Table 1. The variation of skin friction coefficient *Cf*_*x*_ for different values of *δ*, *n*, and *Gm* is portrayed in Fig. 34. It is found that $$ \left|\sqrt{\frac{n+1}{2}}\left(1+\raisebox{1ex}{$1$}\!\left/ \!\raisebox{-1ex}{$\beta $}\right.\right){f}^{{\prime\prime} }(0)\right| $$ increases with *n* while reduces as *δ* and *Gm* increases. The influence of heat transfer rate $$ \left[\sqrt{\frac{n+1}{2}}\left(1+\raisebox{1ex}{$4$}\!\left/ \!\raisebox{-1ex}{$3$}\right.{R}_d\right){\theta}^{\prime }(0)\right] $$ for *R*_*d*_, *β*, and *δ* is perceived in Fig. 35. It is observed that heat transfer rate higher for higher values of *R*_*d*_, *β* whereas increasing values of *δ* diminish the heat transfer rate.

Figure 36 elucidates the effect of heat transfer rate for various values of *N*_*t*_, *N*_*b*_, and Pr. It is noted that mass transfer rate reduces as *N*_*t*_ and *N*_*b*_ increases while increasing values of Pr enhance the mass transfer rate. It is also observed that higher value of Pr enhances the heat transfer rate significantly. Figure 37 illustrates the influence of Sherwood number for several values of *N*_*t*_, *N*_*b*_, and *R*. It is noticed that stronger *N*_*b*_ promotes a progressive increase in mass transfer rate *φ*′(0) whereas increasing values of *N*_*t*_ and *R* decrease the mass transfer rate. Finally, Fig. 38 demonstrates the influence of mass transfer rate for increasing values of *β*, *Le*, and *R*_*d*_. This figure clears that mass transfer rate higher for higher values of *β*, *Le*, and *R*_*d*_.

## Conclusions

Two-dimensional electrically conducting natural convection flow of Casson nanofluid towards nonlinearly stretching sheet in the presence of chemical reaction and thermal radiation was numerically discussed in this study. Moreover, the effect of slip and convective boundary conditions were also considered. Similarity transformations are employed for the conversion of nonlinear partial differential equations to nonlinear ordinary differential equations. Numerical solutions are found by the Keller box method, and graphical results are obtained through MATLAB software. Results are compared with previous work as a limiting case, and excellent accuracy is achieved with those results. It is found that *β* reduces the fluid velocity whereas dimensionless temperature and nanoparticle concentration increase with an increase in *β*. Increasing values of *n* diminish the fluid velocity, temperature, and nanoparticle concentration. Velocity is observed to be enhanced as *Gr* and *Gm* increased. It is also noted that momentum boundary layer thickness decreases as *δ* increases. The dimensionless temperature and nanoparticle concentration profiles increase with increase in *Bi*_1_ and *Bi*_2_, respectively. Furthermore, it is also noticed that dimensionless temperature and nanoparticle concentration distributions are increasing function of *N*_*t*_.
